# Geriatric Oncology in the Instagram Era: PhotoVoice to Enable Patient-Centered Care and Shared Decision Making

**DOI:** 10.1093/geroni/igab046.092

**Published:** 2021-12-17

**Authors:** Irene Blackberry, Christopher Steer, Tshepo Rasekaba, Kim Young, Nicole Webb, Darren Jayasuriya, Kylie Owen, Mira Kapur

**Affiliations:** 1 La Trobe University, Wodonga, Victoria, Australia; 2 Border Medical Oncology and Haematology, Albury, New South Wales, Australia; 3 UNSW, albury, New South Wales, Australia

## Abstract

Evidence shows that multidimensional assessment of older adults with cancer yields more holistic care and results in better communication about age-related concerns; as well as enables personalised, patient-centered supportive care. Geriatric assessment (GA) captures clinical, physical and psychological factors, with limited opportunity to gather information about the patient’s environment, personal contexts and priorities. We trialed the feasibility and acceptability of geriatric assessment (GA)-guided enhanced supportive care (ESC) among 20 adults aged over 70 years in a regional cancer center. We then studied the impact of the integration of four patient-derived photographs (with PhotoVoice analysis) to this ESC on patient satisfaction with communication with the oncologist regarding age-related concerns and on facilitating empowerment, patient-centered care and shared decision making. The use of PhotoVoice analysis of patient-derived photographs is a novel strategy that can facilitate gathering patient-centered information during the assessment process.

